# Bhutan's Response Against Increasing Number of Stroke (BRAINS): A Hybrid Type II Effectiveness-Implementation Study

**DOI:** 10.7759/cureus.103394

**Published:** 2026-02-10

**Authors:** Yangchen, Jeyaraj D Pandian, Tashi Tenzin, Ruitai Shao, Guru P Dhakal, Nar B Rai, Sonam Yangzom, Kezang Tshering, Ivy A Sebastian, Dorcas Gandhi, Gampo Dorji, Tshenday Wangchuk, Karma Tenzin

**Affiliations:** 1 Department of Medicine, Jigme Dorji Wangchuck National Referral Hospital, Khesar Gyalpo University of Medical Sciences, Thimphu, BTN; 2 Department of Neurology, Christian Medical College and Hospital, Ludhiana, IND; 3 Department of Surgery, Jigme Dorji Wangchuck National Referral Hospital, Khesar Gyalpo University of Medical Sciences, Thimphu, BTN; 4 School of Population Medicine and Public Health, Chinese Academy of Medicine Science/Peking Union Medical College, Beijing, CHN; 5 Department of Emergency Medicine, Central Regional Referral Hospital, Gelephu, BTN; 6 Department of Medicine, Eastern Regional Referral Hospital, Mongar, BTN; 7 Department of Pharmacy, Jigme Dorji Wangchuck National Referral Hospital, Thimphu, BTN; 8 Department of Physiotherapy, Christian Medical College and Hospital, Ludhiana, IND; 9 Department of Health Policy, World Health Organization (WHO) Country Office-Nepal, Kathmandu, NPL; 10 Department of Physiotherapy, Jigme Dorji Wangchuck National Referral Hospital, Thimphu, BTN; 11 Department of ENT, Jigme Dorji Wangchuck National Referral Hospital, Thimphu, BTN

**Keywords:** implementation research on stroke care, physician-led stroke care model, stroke care improvement in low-resource settings, task-shifting, thrombolysis

## Abstract

Introduction

Bhutan faces a growing stroke burden as the third leading cause of death, compounded by delayed hospital arrivals, fragmented care, and near-absent thrombolysis due to systemic gaps in protocols and trained workforce.

Methods

This hybrid type II implementation study (2020-2023) employed the Reach, Effectiveness, Adoption, Implementation, and Maintenance (RE-AIM) framework to evaluate a physician-led stroke care model across Bhutan's referral hospitals. Interventions included capacity-building for non-neurologists, protocol standardization, and stroke unit (SU) establishment. We used an uncontrolled pre-post design, comparing six months of retrospective baseline data (January 2020 to June 2020) to prospective post-intervention data (October 2022 to March 2023).

Results

The introduction of a physician-led stroke care model in Bhutan's national referral hospitals led to measurable gains across all RE‑AIM domains. Adherence to stroke care quality indicators nearly doubled (30%-57%), and unadjusted in-hospital mortality decreased substantially from 29% to 9% post‑intervention (adjusted odds ratio {aOR}, 0.65; 95% CI, 0.50-0.85). However, the causal interpretation of the mortality reduction is limited by the lack of adjustment for baseline stroke severity. The sustainability of the intervention faced challenges from high staff attrition (55%), affecting protocol fidelity. This highlighted the need for systemic workforce retention strategies and sustained leadership to ensure long-term maintenance.

Conclusion

The findings of the Bhutan's Response Against Increasing Number of Stroke (BRAINS) initiative suggest that key elements of organized stroke care may be successfully adapted to low-resource settings. The outcomes in Bhutan, consistent with experiences in India and South Africa, suggest that models built around dedicated units, trained teams, and standard protocols hold promise for replication. The long-term sustainability of such improvements, however, seems to rely on addressing systemic constraints. The initiative implies that challenges such as workforce retention and limited specialist availability may be addressed by strategies such as training non-neurologist physicians and establishing clear career pathways for nursing staff. Taken together, the initiative's experience supports the inference that with local tailoring, a structured and team-based approach to stroke care could be a viable model for similar low-middle-income country (LMIC) contexts.

## Introduction

Noncommunicable diseases (NCDs) are becoming a growing public health issue in Bhutan with socio-economic progress and rapid urbanization [1]. Stroke, in particular, has emerged as a devastating condition, ranked as the third leading cause of death nationwide [2].

The Royal Government of Bhutan provides free universal healthcare to the citizens through its extensive network of hospitals and primary healthcare centers, but critical gaps persist in stroke care delivery. A comprehensive five-year study (2011-2015) conducted at Jigme Dorji Wangchuck National Referral Hospital (JDWNRH) highlighted several important trends: an increasing number of stroke admissions (637); notably, 15% of these cases involved patients under the age of 45 [3]. The trend mirrors the situation of other low-income and low-middle-income countries (LMICs), where stroke poses a significant health burden [4].

Most significantly, not a single ischemic stroke patient admitted during this time period received intravenous (IV) thrombolytic therapy despite the availability of thrombolytic medicines in the hospital. This treatment gap arose from multiple challenges, including the lack of awareness about stroke symptoms, delays in seeking care, and the lack of trained health workers and stroke units (SUs). Furthermore, the country's healthcare workforce faced critical shortages with only a few clinicians and nurses trained in acute stroke care management.

Strong evidence demonstrates the effectiveness of organized stroke care systems, including establishing stroke units and offering timely reperfusion therapies [5]. Alternative models of stroke care have been developed and successfully implemented in low-resource settings [6]. Physician-led stroke care initiatives in neighboring India (Tezpur and Ludhiana) have shown promising results, including reduced hospital stay, fewer in-hospital complications, and improved outcomes for stroke patients [6,7]. Drawing inspiration from these lessons, Bhutan launched the Bhutan's Response Against Increasing Number of Stroke (BRAINS) initiative through a collaborative partnership between the Royal Government of Bhutan, World Health Organization South East Asia Region Office (WHO-SEARO), and Christian Medical College Ludhiana (CMCL), India.

This implementation research aimed to transform stroke care across Bhutan's three major referral hospitals: Jigme Dorji Wangchuck National Referral Hospital (JDWNRH) in the capital city of Thimphu, Central Regional Referral Hospital (CRRH) in Gelephu, and Eastern Regional Referral Hospital (ERRH) in Mongar.

Objectives

Primary Objective

Implementation outcomes: The primary objective is to evaluate the process of implementing a standardized stroke care bundle across three national referral hospitals using the Reach, Effectiveness, Adoption, Implementation, and Maintenance (RE-AIM) framework. The primary implementation outcomes were the "Reach" of the intervention to the target population, "Adoption" and "Implementation fidelity," and "Maintenance" of a standardized stroke care bundle by the clinical teams.

Secondary Objective

Effectiveness outcomes: The secondary objective is to assess the association between the implementation of the care bundle and subsequent changes in patient-level clinical outcomes (stroke unit admission, the initiation of rehabilitation within 48 hours, the administration of thrombolytic therapy for eligible ischemic stroke cases, swallow assessment, rates of aspiration pneumonia, and in-hospital mortality).

## Materials and methods

Study setting and design

The study was conducted in Bhutan's three tertiary referral hospitals, where CT and ICU facilities were available but had no stroke unit (SU) prior to the intervention, and stroke care was delivered by internists in the absence of neurologists, with neurosurgical services available at only one center (JDWNRH). The Institutional Review Board of Khesar Gyalpo University of Medical Sciences of Bhutan issued approval IRB/Approval/PN21-033/2021-22/519.

This is a hybrid type II effectiveness-implementation study designed to evaluate both clinical outcomes and the implementation processes of stroke care. Hybrid type II designs are suitable when there is already evidence of clinical benefit, as it allows for the assessment of both clinical impact and the contextual factors that affect adoption and feasibility in the real world. A concurrent control group was not feasible because national policy mandated that the intervention be implemented across the country's three referral hospitals to improve stroke care, and withholding the intervention was neither ethical nor practically acceptable. Therefore, a pre-post design was adopted, with analyses adjusted for age, sex, stroke type, and comorbidities to strengthen internal validity. The implementation process was structured and evaluated using the RE-AIM framework [8].

Stakeholder engagement

Stakeholders were engaged across the entire healthcare system. Semi-structured interviews were conducted with 56 healthcare workers (physicians, nurses, physiotherapists, radiologists, and CT technicians) involved in stroke care, as well as hospital administrators and health ministry officials.

Clinical data collection

Data collection occurred in two phases. The pre-intervention phase (January 1, 2020, to June 30, 2020) involved a retrospective review of patients' case record forms for all stroke admissions. The post-intervention phase (October 1, 2022, to March 31, 2023) used prospective data collection with standardized case report forms capturing stroke characteristics, imaging, treatment, complications, and clinical outcomes from patients' case record forms. Data were entered into a secure electronic database. Clinical care was directly observed at the study centers to assess facility readiness. A comprehensive list of data collection manuals, including stroke care protocols (IV tissue plasminogen activator {tPA} monitoring, Fever-Sugar-Swallow {FeSS} protocol, swallow assessment form, and case record forms), has been listed in the Appendices and will be provided if needed for review.

Inclusion criteria were based on the WHO definition of stroke as a focal (or at times global) neurological impairment of sudden onset, and lasting 24 hours (or leading to death) and confirmed by imaging (CT/MRI scan), in patients aged >18 years. Exclusions included subarachnoid hemorrhage, central nervous system (CNS) neoplasms, CNS vasculitis, CNS Infections, and traumatic brain injury.

Aspiration Pneumonia Diagnosis

Diagnosis required a new infiltrate on chest radiograph plus at least two of the following: fever (>38°C), leukocytosis, purulent sputum, or a documented positive sputum culture, in a patient with a clinical suspicion of aspiration.

Data analysis

Quantitative Analysis

Descriptive statistics were used to characterize the patient population. Continuous variables were stated as mean ± standard deviation or median (interquartile range). Categorical variables are depicted as count (percentage). To evaluate the intervention's impact, we compared pre- to post-intervention outcomes using chi-square tests for categorical variables and Mann-Whitney U tests for continuous variables. A logistic regression model was built to assess the association between the intervention period (post versus pre) and in-hospital mortality. Due to the absence of National Institutes of Health Stroke Scale (NIHSS) scores in the pre-intervention cohort, the model was adjusted for available covariates: age, sex, stroke type (ischemic versus hemorrhagic), and comorbid conditions. The model's fit was assessed using the Hosmer-Lemeshow test. All analyses were conducted in R version 4.2.1 (R Foundation for Statistical Computing, Vienna, Austria), with a significance level of p < 0.05.

Qualitative Analysis

**Figure 1:**
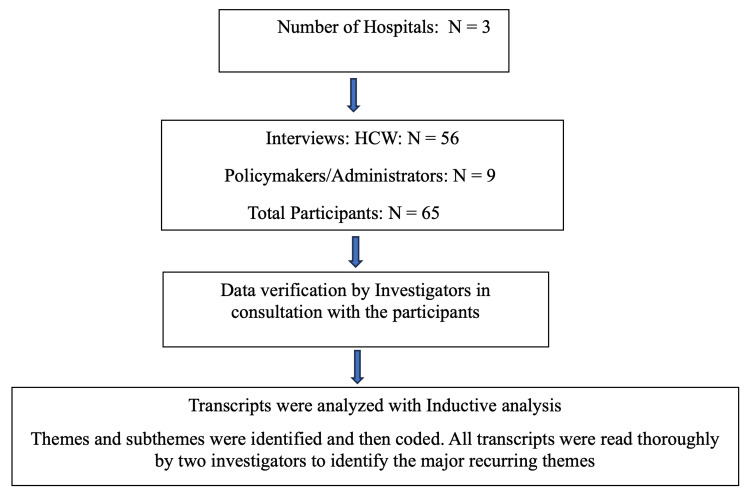
Qualitative data analysis steps HCW: healthcare worker

Interviews were recorded, transcribed verbatim, and anonymized. We employed a framework analysis approach, using the seven key domains from Flottorp et al. as an initial coding framework [9]. Two researchers independently coded a subset of transcripts to develop a consensus codebook. The remaining transcripts were coded by one researcher and reviewed by the other. Emerging themes were discussed and refined within the research team to ensure consensus and thematic saturation. Figure 1 describes the steps in the qualitative data analysis.

Table 1 lists the intervention components focused on four areas: training healthcare workers in acute stroke management (including IV tPA drug alteplase administration), implementing standardized protocols, establishing dedicated stroke units, and creating rapid triage pathways.

**Table 1:** Interventions carried out during the implementation research JDWNRH, Jigme Dorji Wangchuck National Referral Hospital; ERRH, Eastern Regional Referral Hospital; CRRH, Central Regional Referral Hospital; NIHSS, National Institutes of Health Stroke Scale; mRS, modified Rankin scale; FeSS, Fever-Sugar-Swallow; IV, intravenous; CMC, Christian Medical College; ADLs, activities of daily living; IADLs, instrumental activities of daily living; SU, stroke unit; TIA, transient ischemic attack

Serial number	Interventions	Month of completion	Participants
1	Training of clinicians and nurses of JDWNRH, ERRH, and CRRH on evidence‑based stroke care: NIHSS/mRS stroke classification and mechanism (clinical and radiological), hyper-acute stroke management including IV thrombolysis, blood pressure management, swallow assessment, FeSS protocol, prevention and management of complications, and secondary prevention/discharge planning	October 2022 (duration: three months with Zoom and Google Classrooms)	Physicians, 19; nurses, 22; physiotherapists: nine; and CT technicians, six
2	Training of the rehabilitation team at the three hospitals. The physiotherapy team from CMC Ludhiana, India, trained in evidence‑based stroke assessment modules (sensory, motor, functional, cognitive, and perceptual assessments). Evidence‑based stroke rehabilitation modules on consensus‑based definitions and stages of motor recovery and temporal profile of recovery post-stroke. Upper extremity motor and sensory retraining, including hand rehabilitation, gait and balance rehabilitation, acute-phase care, chest and pulmonary care post‑stroke, sensory rehabilitation post‑stroke, retraining of ADLs and IADLs, prevention of secondary complications, cognitive rehabilitation, and introduction to speech and language therapy	October 2022 (duration: three months with Zoom and Google Classrooms)	Physiotherapists of JDWNRH, ERRH, and CRRH
3	Strategy to enhance the system adoption of evidence‑based stroke care guidelines. Familiarization with the importance of the adoption of evidence‑based stroke protocols to improve stroke care, through meetings and presentations. Establish six‑monthly reporting of key quality indicators of stroke care in the three hospitals to the Quality Assurance Division, Ministry of Health, on a routine basis	October 2022 (duration: three months with Zoom and Google Classrooms)	Secretary of the Ministry of Health, Department of Public Health officials, and medical and nursing superintendents of the three hospitals
4	Strategy to enhance the adoption of evidence‑based stroke care guidelines at the hospital and healthcare professional level. Reorganized the existing infrastructure by designating six beds as SU in the Department of Medicine, JDWNRH, and two beds each in the Department of Medicine at ERRH and CRRH as SU. Implemented the following stroke care protocols in the newly established SUs: IV thrombolytic therapy for eligible ischemic stroke patients; start aspirin within 48 hours of admission in ischemic stroke or TIA; control of blood pressure, blood sugar, fever, and dysphagia screening as per FeSS protocol; prophylaxis for the prevention of deep vein thrombosis (DVT); monitoring and managing medical complications; and the initiation of secondary prevention medications and health education. Strengthened the physician‑led model of stroke care at the three hospitals through meetings with the stroke teams of the three hospitals	October 2022 (duration: three months with Zoom and Google Classrooms)	Administrators of the three hospitals, Department of Medicine staff, and Department of Emergency staff

## Results

A total of 264 stroke patients (64% men; n = 169, median age 64) were admitted to the three hospitals (pre-intervention sample size = 110; post-intervention sample size = 154) over a period of one year, with maximum admissions at the national referral hospital (172 in JDWNRH). Ischemic stroke accounted for 61% of the cases (n = 162). Hypertension was the predominant risk factor (78%, n = 206), with 25% of those patients having discontinued antihypertensive treatment before admission. The intervention's impact was evaluated using the RE-AIM framework (Table 2), drawing on quantitative indicators and qualitative interviews.

**Table 2:** RE-AIM framework to assess the effectiveness of the interventions in improving stroke care across three hospitals in Bhutan ERRH, Eastern Regional Referral Hospital; CRRH, Central Regional Referral Hospital; JDWNRH, Jigme Dorji Wangchuck National Referral Hospital; IV, intravenous; tPA, tissue plasminogen activator; WHO, World Health Organization

RE-AIM dimension	Definition	Indicator	Data sources
Reach	Proportion and number of healthcare workers looking after stroke cases. Number of stroke patients treated in the stroke unit. Barriers and facilitators	Healthcare workers trained: 56 (physicians, 19; nurses, 22; CT technicians, six; and rehabilitation technicians, nine). Number of stroke patients treated in the stroke unit: 54 (35%). Stakeholder engagement: 17 scheduled group training sessions and five high-level meetings with hospital administrators held to ensure system-wide reach and address barriers. Barriers and facilitators: qualitative data analysis identified key themes influencing the implementation of the stroke care protocol	Training register, interviews, emails, and meeting notes
Effectiveness	Primary and secondary outcomes	Healthcare system level: stroke units established with 10 beds across three sites (ERRH, two; CRRH, two; and JDWNRH, six). Stroke teams formed, including a physician, nurse, physiotherapist, and radiologist. Patient outcome level (pre versus post): unadjusted mortality decreased by 20% (29%-9%, p < 0.001). Aspiration pneumonia decreased by 14% (39%-24.7%, p = 0.005). No complications during hospital stay increased by 13% (34%-47%, p = 0.026). Key quality indicators of the process of care: IV tPA administration introduced; 7% of ischemic stroke patients received treatment (p = 0.02). Swallow assessment increased by 75% (10%-85%, p < 0.001)	Training register and WHO key quality indicators obtained from patient files
Adoption	Adoption of evidence-based stroke care protocols at the healthcare worker and organizational level	Policy level: Ministry of Health formally recognized the need and facilitated adoption by issuing an official circular mandating the use of evidence-based stroke care guidelines. Organizational level: hospital administrators and medical superintendents/nursing supervisors at all three sites coordinated and facilitated adoption. Clinical level: a high proportion of stroke patients received care according to new protocols, evidenced by improvement in key quality indicators	Office order of the Secretary of Health, training records, and patient case records
Implementation	Fidelity to evidence-based stroke care protocols	Fidelity to protocol: high adherence documented via post-intervention audit of key quality indicators. Supervision and monitoring: multidisciplinary team review meetings held on a six-monthly basis. Data-driven management hindered: a critical shortage of dedicated staff prevented the submission of key quality indicator data to the Ministry of Health Quality Assurance Division	Patient case record forms, meetings held, and data collection by the Quality Assurance Division
Maintenance	Institutionalization and sustained practice	Institutionalization: stroke care protocols integrated into hospital policy. Establishment of 10 permanent stroke unit beds and a mandate from the Ministry of Health indicate institutional commitment. Sustained practice: data collected over six months shows adherence to key protocols. Culture: interviews with healthcare professionals, administrators, and policymakers reveal a cultural shift toward multidisciplinary, protocol-driven stroke care	Hospital policy documents, patient data, and interviews

Process of care measures

Significant improvements were observed in key stroke care processes following implementation. Swallowing screening within 24 hours increased from 10 to 85%. The documentation of the NIHSS increased from 0% to 43%. The initiation of rehabilitation within 72 hours increased from 42% to 94%. Intravenous tPA administration increased from 0% (0/55) in the pre‑intervention cohort to 7% (7/105) in the post‑intervention cohort (p = 0.02). Composite adherence to key stroke quality indicators increased from 30% in the pre-intervention to 57% post-intervention.

Though most quality indicators improved, the rate of CT scans obtained within one hour increased only modestly (8%-13%), and guideline-directed anticoagulation prescription rates increased (33%-63%) but remained below the international standard. The comparison of quality indicators before and after the intervention is presented in Table 3.

**Table 3:** Comparison of key quality indicators (KQI) of stroke care before and after the intervention The pre- and post-intervention data are plotted in columns and KQI in rows. Pre-intervention sample size = 110; post-intervention sample size = 154. For therapy-specific indicators (tPA, aspirin, and anticoagulant {ACA}), denominators are restricted to eligible ischemic stroke patients (pre-intervention sample = 55; post-intervention sample = 105) tPA, tissue plasminogen activator; AF, atrial fibrillation; NIHSS, National Institutes of Health Stroke Scale; KPI, key performance indicator

KQI	Pre-intervention	Post-intervention	P-value
Proportion of stroke patients who receive a CT scan within one hour of hospital arrival	8% (9/110)	13% (20/154)	0.256
Proportion of stroke patients who are screened or assessed for swallowing deficits	10% (11/110)	85% (131/154)	<0.001
Proportion of ischemic stroke patients who are treated with tPA	0% (0/59)	6.5% (7/107)	0.023
Proportion of ischemic stroke patients who receive acute aspirin	81% (48/59)	88% (94/107)	0.289
Proportion of stroke patients who are admitted to an acute stroke unit	0% (0/110)	35% (54/154)	<0.001
Proportion of patients who receive mobilization within 72 hours	42% (46/110)	94% (145/154)	<0.001
Proportion of stroke patients in inpatient rehabilitation who are treated on a rehab stroke unit	42% (46/110)	94% (145/154)	<0.001
Percentage of stroke patients in hospital or rehabilitation facility who experience a fall post stroke	0% (0/110)	0% (0/154)	1
Proportion of ischemic stroke patients who are prescribed a statin agent (system indicator: the availability of statin medications in the region)	50% (55/110)	91% (140/154)	<0.001
Proportion of patients with AF prescribed ACA	55% (6/110)	50% (17/34)	1
Proportion of patients with NIHSS	0% (0/110)	43% (66/154)	<0.001
Overall KPI adherence for the hospital	32%	59%	<0.001

Health system and service delivery changes

All three hospitals established stroke units and multidisciplinary stroke teams during the intervention period. Staff interviews reflected the uptake of standardized protocols within stroke units. Stroke service characteristics before and after interventions at each hospital are summarized in Table 4.

**Table 4:** Comparison of stroke services at each hospital before and after intervention

Stroke services	Pre-intervention at Jigme Dorji Wangchuck National Referral Hospital, Thimphu	Pre-intervention at Eastern Regional Referral Hospital, Mongar	Pre-intervention at Central Regional Referral Hospital, Gelephu	Post-intervention at Jigme Dorji Wangchuck National Referral Hospital, Thimphu	Post-intervention at Eastern Regional Referral Hospital, Mongar	Post-intervention at Central Regional Referral Hospital, Gelephu
Stroke unit (SU)	Not available	Not available	Not available	6-bed SU	2-bed SU	2-bed SU
Thrombolytic therapy	Not available	Not available	Not available	Available	Available	Available
Evidence-based stroke care protocols	Not in place	Not in place	Not in place	In place	In place	In place
Access to rehabilitation	Available	Available	Available	Available	Available	Available

Healthcare worker perspectives

Pre-intervention interview revealed the limited awareness of organized stroke care. Physicians reported a lack of experience with thrombolysis and concerns about bleed risk, while nurses described limited familiarity with stroke protocols. Nurses highlighted unmet patient expectations due to communication challenges and high workload pressures. Table 5 presents the pre-intervention interview.

**Table 5:** Pre-intervention interview: main themes and subthemes to emerge from interviews and common perceptions about enablers and barriers to providing evidence-based stroke care NCD, noncommunicable disease; IV, intravenous; tPA, tissue plasminogen activator; PEN, package of essential noncommunicable (NCD) disease interventions; HEARTS, healthy-lifestyle counseling, evidence-based treatment protocols, access to essential medicines and technology, risk-based management, team-based care, and systems for monitoring; SCCI, Service with Care and Compassion Initiative; EMS, emergency medicine service; CSO, civil society organization; CME, continuing medical education

Themes	Subthemes	Perceived enablers	Perceived barriers
National-level stroke care initiatives	Policy support: exists for NCD and stroke prevention awareness	Awareness: policymakers recognize the need to improve stroke care. Primary prevention: NCD division provides standardized PEN HEARTS/SCCI training to all healthcare workers (HCWs)	Strategy: no formal, dedicated national plan for hospital-based stroke care
Stroke services	Acute care, EMS, IV thrombolytic therapy, stroke unit, knowledge on stroke care, patient expectations, and community support	Resources: CT scanners, IV thrombolytic drugs (tPA), ambulances, and aeromedical services. Staff motivation: nursing staff interested in stroke care. Financial support: free transport, healthcare, and food. Community role: stroke foundation (CSO) supports survivors	Lack of protocols and training: no EMS training, no standardized tPA protocols, and a lack of physician confidence. Staff shortages: few neurologists and limited CME. Service gaps: no acute evidence-based stroke services. Poor communication limits patient education. Families desire clearer explanations and community support
Rehabilitation services	Framework for stroke rehabilitation and standardized swallow assessment	Basic framework: neurophysiotherapy units exist in three hospitals for in/outpatients	Lack of continuum of care: no post-discharge system and rare follow-up. Resource gaps: severe shortage of speech therapists and limited swallow assessments outside the central hospital
Infrastructure/human resource (HR)	CT scan, thrombolytic drug, human resource, and stroke unit	Equipment: CT scans and tPA available in three referral hospitals	Untrained staff: HCWs not trained in evidence-based protocols. Absence of stroke units. Underutilized resources: equipment and drugs not used due to the lack of pathways and training

Post-intervention interviews described improvement in workflow, protocol use, and the coordination of patient management. Staff reported greater clarity in role and increased confidence in delivering structured stroke care. Themes and subthemes from these interviews are summarized in Table 6.

**Table 6:** Post-intervention: main themes and subthemes to emerge from interviews and common perceptions about enablers and barriers to providing evidence-based stroke care after implementing interventions NCD, noncommunicable disease; FeSS, Fever-Sugar-Swallow

Themes	Subthemes	Enablers to providing evidence‑based stroke care	Barriers to providing evidence‑based stroke care
National-level stroke care initiatives	Policy support; strategy to improve stroke care in the hospitals	Policy endorsement: formal support from the Ministry of Health via a policy paper mandating evidence‑based stroke care in referral hospitals. Structured training: phased implementation of standardized NCD modules for healthcare workers, focusing on prevention and early care pathways	Programmatic gap: continued absence of a dedicated, centrally managed national stroke program
Stroke services	Acute care EMS, professional development for staff, stroke unit (SU), standardized nursing care in SU, community support for stroke patients, and patient expectations	System implementation: successful establishment of a prehospital EMS system with trained personnel for early stroke recognition and activation. Clinical infrastructure: the creation of functional stroke units (SUs) in three referral hospitals, adhering to FeSS protocols, and administering IV thrombolysis. Enhanced workforce capacity: significant increase in healthcare worker confidence, protocol awareness, and ability to communicate effectively with patients and families. Strong partnerships: effective advocacy and public awareness campaigns led by the Bhutan Stroke Foundation, supported by technical advisors from the trained cohort. Improved communication and patient assurance: training significantly increased healthcare workers' confidence in discussing stroke, leading to more effective patient and family education on disease progression and risk factors	Workforce instability: high staff attrition and turnover necessitate continuous training and a dedicated stroke focal person per hospital to ensure protocol adherence and data collection. Resource limitations: established stroke units (SUs) operate with suboptimal nurse‑to‑patient ratios due to a national nursing shortage. Unequal access: evidence‑based stroke services remain unavailable in many hospitals outside the main referral centers. Geographical and infrastructural challenges: rough terrain and weather often prevent transfer within the treatment window, compounded by the persistent public lack of awareness in recognizing stroke signs. Fragmented care continuum: inconsistent application of secondary prevention, poor patient follow‑up, and low compliance remain significant issues post‑discharge. A lack of community support: a clear need exists for structured, community‑based support systems for patients and their families
Rehabilitation services	Framework for stroke rehabilitation; standardized swallow assessment	Established infrastructure: basic neurophysiotherapy units are operational for inpatients and outpatients in the three referral hospitals. Upskilled nurses: training has enabled nurses to effectively screen for dysphagia, allowing for triage to a specialist speech pathologist	Fragmented post‑discharge care: a critical lack of a coordinated system results in poor patient follow‑up after hospital discharge. Specialist shortage: a severe shortage of speech therapists, particularly in regional hospitals, limits comprehensive swallow assessments and creates treatment delays. General resource constraints: overall limitations in staffing and equipment hinder the delivery of optimal rehabilitation services

Sustainability and ongoing challenges

Despite improvements, several challenges were identified. High attrition rates among trained staff, reaching 55%, affected the continuity of care and protocol adherence. Nursing staff reported a challenge related to the time required for completing stroke-related documentation. Gaps remained in post-discharge care, secondary prevention, and community-based support. Stakeholders emphasized the need for designated stroke leadership roles, such as stroke nurse coordinators, and structured career pathways to sustain long-term sustainability.

## Discussion

Bhutan's experience offers a feasible and replicable model for LMICs: our study shows that a multifaceted approach combining policy directives, structural reforms, and capacity building can improve processes of stroke care despite resource constraints.

The implementation of the stroke unit and pathway was associated with improvements in clinical outcomes: in-hospital mortality and aspiration pneumonia. Though intravenous tPA administration increased modestly, it reached statistical significance in the post‑intervention cohort, and importantly, it demonstrates the successful initiation of thrombolytic therapy in routine practice where it was previously absent. Furthermore, process indicators, such as swallowing screening within 24 hours, the documentation of NIHSS, and the initiation of rehabilitation within 48 hours, improved substantially. These quantitative gains were supported by qualitative feedback from staff who reported greater protocol adherence, improved efficiency, and organized patient management within the stroke unit. These findings are consistent with evidence from Cochrane reviews, which have shown that the establishment of stroke units reduces overall poststroke mortality [10].

The contribution of this work lies not in conceptual novelty but in its detailed, documented account of implementing and assessing a stroke care bundle, evaluated through the RE-AIM framework, within Bhutan, an LMIC with a free, public healthcare model. The initiative's outcomes build upon lessons from similar implementation efforts across the Global South.

Most LMICs in Southeast Asia face a limited availability of neurologists or stroke specialists [11]. However, successful models, such as the physician-led stroke care model "Tezpur Model" in rural India, align closely with the stroke care pathway implemented in Bhutan [6]. Both studies demonstrate that neurologists are not an absolute prerequisite for implementing stroke care bundles; instead, training motivated physicians that evidence-based protocols can significantly improve outcomes in remote settings. Our study also corroborates the findings of de Villiers et al. in South Africa, which showed that multidisciplinary care (in their case, involving a team of doctors, nurses, and therapists) significantly improved functional outcomes and reduced mortality, even without advanced technology [11]. The formation of stroke teams in our hospitals was an important intervention, moving away from a fragmented approach to a coordinated, team-based model that is essential for comprehensive stroke care.

However, our study also revealed systemic challenges that mirror the systematic review by Pandian et al. [12]; workforce attrition is the biggest challenge threatening sustainability. The departure of over half of the trained staff is similar to the crisis faced by many LMIC health systems and highlights an important finding that training programs alone are insufficient without strong retention strategies such as career incentives and professional recognition.

Implications for scale-up

The study suggests several strategies for sustaining and scaling stroke care in LMICs.

Workforce Retention

Career pathways, professional recognition, and targeted incentives are essential to protect investments in human resources.

Integration With NCD Programs

Embedding stroke care into broader national NCD initiatives can enhance funding, political support, and the holistic management of risk factors.

Community Awareness and Early Treatment

Training community health workers and implementing public awareness campaigns are critical to reducing delays in hospital arrival and improving outcomes.

Context-Specific Adaptation

Replication in other LMICs requires tailoring to local infrastructure, staffing stability, imaging availability, and population awareness while maintaining the core components of protocols, training, and stroke units.

Limitations and generalizability

Our study has several important limitations.

Differences in data collection methods may have introduced bias. The pre-intervention cohort relied on retrospective chart review, making it vulnerable to incomplete documentation, whereas the post-intervention cohort was prospectively collected, potentially enhancing data quality and exaggerating observed improvements in process indicators. Furthermore, although regression analyses were adjusted for age, sex, stroke type, and comorbidities, variables consistently available across both cohorts, residual confounding remains possible. In particular, the absence of NIHSS scores in the pre-intervention cohort limited our ability to adjust for stroke severity, and the observed mortality reduction should therefore be interpreted with caution.

Implementation was challenged by a high attrition rate of trained healthcare workers, which threatened the fidelity and consistency of protocol delivery. The study period (2020-2023) overlapped with the COVID-19 pandemic, which may have independently affected stroke care pathways, hospital admissions, and patient outcomes. While we adjusted for available case-mix variables, unmeasured system-wide changes remain a potential confounder.

Documentation Bias

Improved documentation can occur during the implementation of structured protocols. The observed increases in adherence to process measures (e.g., documented dysphagia screening) may partly reflect more rigorous recording rather than a true change in clinical practice. We attempted to address this by ensuring data collections were distinguished between the documentation of an action and evidence that the action was performed.

Regarding generalizability, the study was conducted only in three referral hospitals with CT imaging capabilities, which limits its direct applicability to smaller primary care facilities. The model is most promising for similar LMIC settings that have at least basic imaging and a core group of stroke teams. However, generalizability is highly dependent on context. The success in Bhutan was facilitated by strong ministerial support and a free public health system. Other regions may face greater challenges related to healthcare financing, patient costs for thrombolytics, or more fragmented referral networks. Future adaptations must conduct contextual analyses to address local resource gaps and barriers.

## Conclusions

The findings from the BRAINS initiative suggest that key components of organized stroke care, such as dedicated stroke units, trained multidisciplinary teams, and standardized protocols, may be effectively implemented in low-resource settings. This aligns with evidence from comparable contexts in India and South Africa, reinforcing the potential transferability of such models.

The study further suggests that sustaining these improvements is dependent upon addressing systemic challenges, notably workforce retention and the limited availability of neurologists. Evidence from this initiative implies that strategies such as specialized training for non-neurologist physicians and the establishment of career pathways for stroke nurses could support long-term viability. Overall, the experience in Bhutan supports the inference that a structured, team-based approach to stroke care can be adapted and replicated in similar LMIC contexts, provided implementation is tailored to local health system capacities.

## Figures and Tables

**Figure 2:**
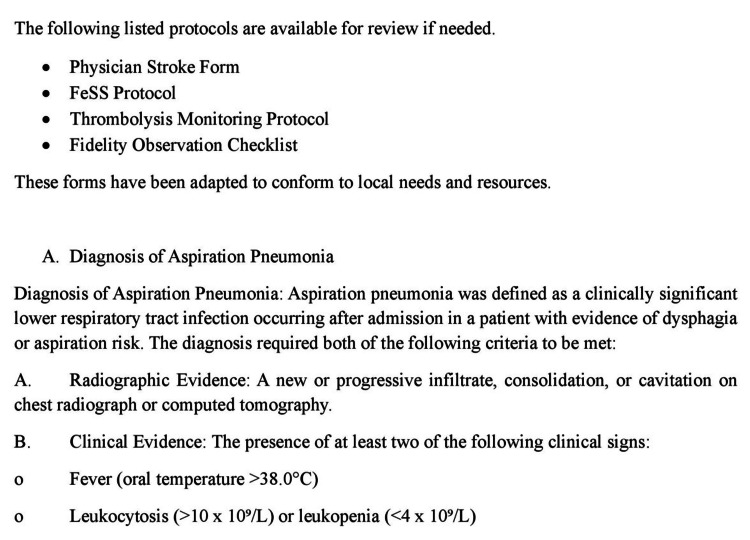
List of protocols available for review and the operational definition of aspiration pneumonia FeSS: Fever-Sugar-Swallow

## Appendices

Figure 2 shows the comprehensive list of data collection manuals, including stroke care protocols.
